# Charge-Transfer Process in Surface-Enhanced Raman Scattering Based on Energy Level Locations of Rare-Earth Nd^3+^-Doped TiO_2_ Nanoparticles

**DOI:** 10.3390/nano11082063

**Published:** 2021-08-14

**Authors:** Zihao Zhao, Xiang Zhao, Mu Zhang, Xudong Sun

**Affiliations:** 1 Key Laboratory for Anisotropy and Texture of Materials (Ministry of Education), Northeastern University, Shenyang 110819, China; 1970495@stu.neu.edu.cn (Z.Z.); zhangm@mail.neu.edu.cn (M.Z.); 2 Laboratory of Advanced Ceramics, Foshan Graduate School, Northeastern University, Foshan 528311, China; 3 State Key Laboratory of Rolling and Automation, Northeastern University, Shenyang 110819, China

**Keywords:** surface-enhanced Raman scattering, charge-transfer, Nd-TiO_2_, 4-Mpy, energy level location

## Abstract

Surface-enhanced Raman scattering (SERS) for semiconductor nanomaterial systems is limited due to weak Raman signal intensity and unclear charge-transfer (CT) processes for chemical enhancement. Here, rare-earth element neodymium-doped titanium dioxide (Nd-TiO_2_) nanoparticles (NPs) were synthesized by the sol–gel method. The characterizations show that the doping of Nd ions causes TiO_2_ NPs to show an increase in the concentration of defects and change in the energy level structure. The CT process between Nd-TiO_2_ NPs substrate and probe molecule 4-Mercaptopyridine (4-Mpy) was innovatively analyzed using the relative energy level location relationship of the Dorenbos model. The SERS signal intensity exhibits an exponential enhancement with increasing Nd doping concentration and reaches its optimum at 2%, which is attributed to two factors: (1) The increase in the defect concentration is beneficial to the CT process between the TiO_2_ and the probe molecule; (2) the introduction of 4*f* electron orbital energy levels of rare-earth ions created unique CT process between Nd^3+^ and 4-Mpy. Moreover, the Nd-TiO_2_ NPs substrate shows excellent SERS performance in Raman signal reproducibility (RSD = 5.31%), the limit of detection (LOD = 10^−6^ M), and enhancement factor (EF = 3.79 × 10^4^). Our work not only improves the SERS performance of semiconductor substrates but also provides a novel approach to the development of selective detection of probe molecules.

## 1. Introduction

Due to the high sensitivity, good selectivity, non-destructiveness, and information about the structure of adsorbed molecules, surface-enhanced Raman scattering (SERS) has attracted widespread interest [1,2,3]. Meanwhile, SERS has a high enhancement factor (up to 10^8^ times or higher), realizing trace detection analysis and even single-molecule-level analysis [4,5]. The mechanisms of SERS enhancement are the electromagnetic mechanism (EM) and chemical mechanism (CM) [6,7]. The EM is related to the localized surface plasmon resonance (LSPR) of noble metal nanoparticles (NPs), such as gold, silver, and copper [8,9]. Due to LSPR, the electric field intensity on the surface of the noble metal NPs is significantly increased, so when the probe molecules are close to the surface, strong Raman scattering will be generated [10]. Studies have shown that the EM has nothing to do with the type of probe molecules, it is a long-range effect, and the Raman signal of molecules within about 2 nm from the substrate surface can be enhanced [11]. The CM is based on the new chemical bonds formed by the adsorption of probe molecules onto the substrate [12,13]. The reasons for enhancement are: (1) The charge-transfer (CT) process between the molecules and substrate; (2) the increase in the polarizability of the probe molecules; (3) electron–hole pairs produce an electronic resonance effect, and CT is mainly attributed to them [14,15,16]. Moreover, the CM is a short-range effect, which usually only occurs on the first layer of adsorbed molecules [17,18]. The traditional noble metal SERS substrate has been widely developed due to its high sensitivity, etc. However, due to the high price, low biocompatibility, and poor molecular selectivity of noble metal substrate [19,20], researchers began to develop non-metal substrate systems, such as semiconductors, graphene, and carbon nanotubes (CNTs) [21,22,23]. The SERS substrate of semiconductor nanomaterials can produce a CT process. The energy level matching between the probe molecules and SERS substrates is the key to analyzing the CT. It exhibits unique properties compared with noble metal SERS substrates, such as a simple preparation process, controllable bandgap, excellent SERS signal stability, high molecular selectivity, and outstanding biocompatibility [24]. Yamada et al. [25] first discovered the enhancement of the Raman signal on pyridine molecules adsorbed on NiO. Yang et al. [26] synthesized TiO_2_ NPs (8–13 nm) by the sol–hydrothermal method, compounded them with sulfhydryl molecules (4-MBA, 4-Mpy, and 4-PATP), and the Raman signal was significantly enhanced. Xue et al. [27] studied the influence of crystallite size and quantum confinement on the SERS performance of TiO_2_ NPs, and their ability to adsorb 4-MBA of different sizes, and the Raman signal reached a maximum when the TiO_2_ NP size was 10.9 nm. Lombardi et al. [28] used ZnO nanocrystals to detect 4-Mpy solution with a limit of detection (LOD) of 10^−5^ M and an enhancement factor (EF) of 1000. However, the development of semiconductor substrates was limited by their low EF, whose use in analysis and detection is still challenging to carry out. Therefore, it is of great importance to design semiconductor SERS substrate with an outstanding EF and LOD.

Researchers usually enhanced the SERS signals of semiconductor nanomaterials through ion doping, heterogeneous recombination, morphology, and structure design. Among them, ion doping is a highly effective method, increasing the defect concentration and adjusting the bandgap of SERS substrates. Zhou et al. [29] reported a substantial enhancement of the SERS signal by near-infrared (NIR) light illumination with a hybrid system consisting of silver and silver-doped titanium dioxide and adsorbed 4-MBA (4-MBA/Ag/Ag-doped TiO_2_), which was attributed to the higher crystallinity of the substrate and the promoted CT. Tian et al. [30] used 2D SnSe_2_ nanosheets as SERS substrates, which essentially broke the limitations of 2D semiconductors for SERS applications and obtained a Raman signal intensity of R6G that was 1.3 to 1.7 times higher than that of pure SnSe_2_. Yang et al. [31] developed a novel sensitive Mo-doped Ta_2_O_5_ semiconductor substrate by the “coupled resonance” effect, which can detect methyl violet (MV) molecules up to 9 × 10^−9^ M. This SERS enhancement effect can be attributed to (i) MV molecular resonance, (ii) CT resonance between MV and Ta_2_O_5_, and (iii) electromagnetic enhancement around the gap and tip of the substrate. Li et al. [32] investigated the effect of Ga doping on ZnO nanoparticles and 4-MBA, and the results showed that the bandgap of ZnO could be narrowed by Ga doping, which in turn affects the CT resonance process and ultimately enhances the intensity of the Raman signal. In particular, rare-earth elements are rarely used for the doping of SERS substrates, and because of their rich energy level structure and unique optoelectronic properties, they can be used to expand SERS applications.

In this work, Nd-TiO_2_ NPs as SERS substrate were prepared via a simple sol–gel method with different Nd doping concentrations (atom% = 0%, 1%, 1.5%, 2%, 2.5%, and 3%). The results show that the surface morphology, concentrations of defects, and energy level structure of the substrates were changed with an increasing Nd/Ti ratio concentration compared with pure TiO_2_ NPs. The SERS signal intensity is greatly enhanced and reaches its optimum at a doping concentration of 2%, which is the chemical enhancement mechanism triggered by a unique CT process that occurs between the Nd-TiO_2_ and 4-Mpy probe molecule. This work specifically describes the CT process between the rare-earth ions and the probe molecule by the Dorenbos model [33], and which is further validated by SERS analysis under different laser excitation wavelengths. The substrate offers excellent SERS performance, and also facilitates the exploration of the selective detection of various probe molecules in SERS applications.

## 2. Experimental Section

### 2.1. Materials

All water was distilled and purified to Milli-Q quality. Titanium butoxide (C_16_H_36_O_4_Ti, 98%), neodymium nitrate hexahydrate (Nd(NO_3_)_3_·6H_2_O, 99.0%), 4-Mercaptopyridine (C_5_H_5_NS, 96%), and ethanol (C_5_H_5_OH, ≥99.7%) were purchased from Shanghai Aladdin Biochemical Technology Co., Ltd. (Shanghai, China).

### 2.2. Preparation of Nd-TiO_2_ NPs

The Nd-doped TiO_2_ NPs SERS substrates were prepared by the sol–gel method. First, C_16_H_36_O_4_Ti and Nd(NO_3_)_3_·6H_2_O were dissolved in 13.4 mL of ethanol with 0.4 mL of acetylacetone with atomic ratios of 0.5%, 1%, 1.5%, 2%, 2.5%, and 3% under stirring for 20 min. Then, a mixed solution of 0.16 mL HCl and 2.02 mL deionized H_2_O was added into the mixed solution under stirring to carry out hydrolysis, and golden yellow transparent sol was obtained by continuously stirring for 100 s. Finally, the as-prepared sol was dried at 80 °C for 24 h in an oven and then calcined for 500 °C with a heating rate of 2 °C·min^−1^ and was held at that temperature for 1 h. Meanwhile, the pure TiO_2_ substrate was also prepared with the same method.

### 2.3. Characterization

X-ray diffraction (XRD, Model Smartlab; Rigaku Ltd., Tokyo, Japan) using graphite monochromatic copper radiation (Cu Kα) (λ = 0.15406 nm) was used to analyze the crystal structure and phase composition within the term of 2*θ* in the range 10–90° at a speed rate of 4°·min^−1^. The morphologies and microstructure of prepared samples were observed using field emission scanning electron microscopy (FE-SEM, Model JSM-7001F, JEOL, Tokyo, Japan) under the condition of 15 kV and transmission electron microscopy (TEM, Model JSM-2000FX, JEOL, Tokyo, Japan) operating at 200 kV.

The surface component and binding energy were determined using an X-ray photoelectron spectrometer (XPS, Model Axis supra, Shimazu-Kratos Analytical, Manchester, UK) with monochromatized Al Kα X-ray radiation. The optical absorption spectra were recorded on a UV–Vis–NIR spectrophotometer (UV–Vis, Model UV-3600 Plus, Shimadzu, Kyoto, Japan) with BaSO_4_ as a reference.

### 2.4. SERS Analysis

SERS signals were detected by a Raman spectrometer (Raman, Model XploRA Plus, HORIBA Scientific, Kyoto, Japan) with 532, 638, and 785 nm He-Ne lasers as excitation sources, the diffraction grid was set as 1200 gr·mm^−1^, the data acquisition was set as double 15 s accumulations for the samples, a laser spot area with a diameter of ~0.72 μm was probed using a 100× objective lens, and incident power at the sample was 1.0 mW. 4-Mpy was used to be probe molecules. In order to find the optimal Nd doping concentration of TiO_2_ substrates, 20 mg of Nd-TiO_2_ NPs (atomic ratio = 0.5%, 1%, 1.5%, 2%, 2.5%, and 3%) mixed with 5 mL 4-Mpy (10^−2^ M) ethanol solution under 1 h ultrasonic dispersion was used to ensure absolute adsorption and dried under ambient conditions for 24 h. Meanwhile, the reproducibility, sensitivity, and LOD of the SERS substrates were assessed using the same method. A normal Raman signal of 10^−2^ M 4-Mpy ethanol solution without substrate was also acquired to calculate EF.

## 3. Results and Discussion

### 3.1. XRD Analysis

**Figure 1:**
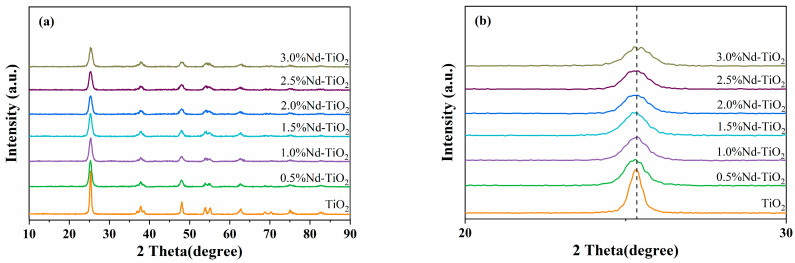
(**a**) XRD patterns of the TiO_2_ and Nd-TiO_2_ NPs with different Nd^3+^ concentrations. (**b**) The enlarged XRD spectra in the plane peak at (101) plane.

XRD was carried out to investigate the crystal structure and degree of crystallinity of the prepared Nd-TiO_2_ NPs with different doping concentrations. The XRD patterns of the samples along with corresponding enlarged spectra in the plane peak at the (101) plane are shown in Figure 1. Figure 1a reveals that all samples have the single anatase phase, which is well indexed as reported in JCPDS file No. 21-1272 [34]. No diffraction peak of neodymium oxides could be detected, indicating that Nd doping concentration was extremely low and out of detection limits of the XRD instrument or part of the Nd ions doped in the TiO_2_ lattice [35]. Additionally, as the doping content of Nd ions increased, the relative intensity of the peak decreased significantly, the full width at half-maximum of the (101) peak greatly increased, and the average particle size of Nd-TiO_2_ at the (101) plane can be estimated according to Scherrer equation:

$$D=\frac{K\lambda }{\beta \cos\theta }$$

where *D* is crystalline size, λ the wavelength of X-ray radiation (0.1541 nm), *K* is a constant usually taken as 0.89, β is the peak width at half-maximum height, and θ is the diffraction angle. The average particle size of samples was calculated to be from about 16.4 nm to 8.2 nm with the increment in Nd doping, which is because the introduction of Nd hinders the crystal growth of TiO_2_ NPs. Furthermore, an enlarged peak of the XRD spectra at (101) is shown in Figure 1b [36]. It can be seen that there is no obvious shift of the main peak at (101) as Nd doping in TiO_2_ though the ion radius of Nd^3+^ (0.0983 nm) is much larger than that of Ti^4+^ (0.0605 nm), which might be due to the coeffect of the ionic radius difference between Nd^3+^ and Ti^4+^ and the reduced particle size [37,38].

### 3.2. SEM and TEM Analysis

**Figure 2:**
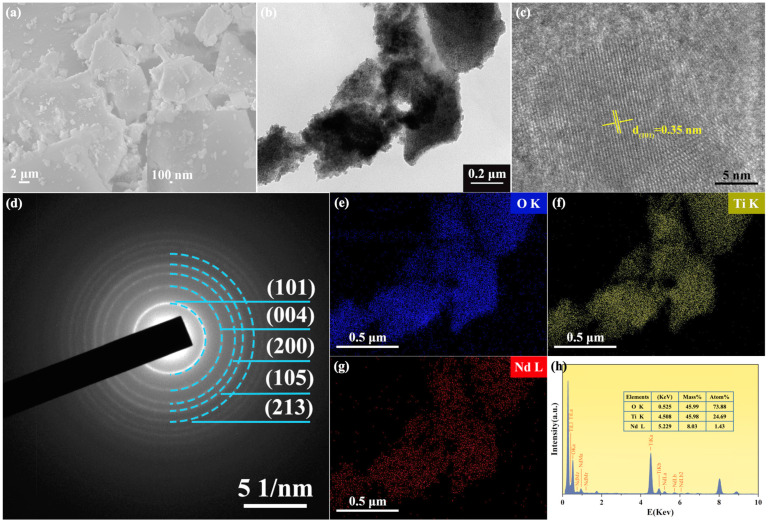
Characterization analysis of the 2%Nd-TiO_2_ NPs. (**a**) SEM and (**b**) TEM images; (**c**) HRTEM image showing lattice space; (**d**) SAED pattern mainly showing the (101), (004), (200), (105), and (213) crystal faces; the element mapping of (**e**) O, (**f**) Ti, and (**g**) Nd; (**h**) EDS spectrum.

The characterization analysis of the 2%Nd-TiO_2_ (Figure 2) and TiO_2_ (in the Supporting Information, Figure S1) NPs was carried out by SEM and TEM. As shown in Figure 2a,b, it can be seen that the samples are agglomerated in a sharp but irregular block shape and some nanoparticles are present on the surface after Nd doping. The high-resolution transmission electron microscope (HRTEM) image of 2%Nd-TiO_2_ NPs shows clear lattice fringes with interplanar crystal spacing of 0.35 nm, corresponding to the (101) crystal plane of the anatase TiO_2_, which is virtually unchanged from that of TiO_2_ (Figure S1c), further confirming that only a small amount of neodymium enters the TiO_2_ lattice. In the selective area electron diffraction (SAED) pattern, it was further verified that the samples are polycrystalline structures of anatase TiO_2_ with (101), (004), (200), (105), and (213) concentric diffraction rings, which matches previous XRD analyses. The elemental mapping patterns (Figure 2e–g) and energy dispersive spectrum (EDS) show that Nd elements with minimal doping are homogenously distributed in the TiO_2_ NPs, and unmarked peaks are copper ions from the copper grid of the sample holder.

### 3.3. XPS Analysis

**Figure 3:**
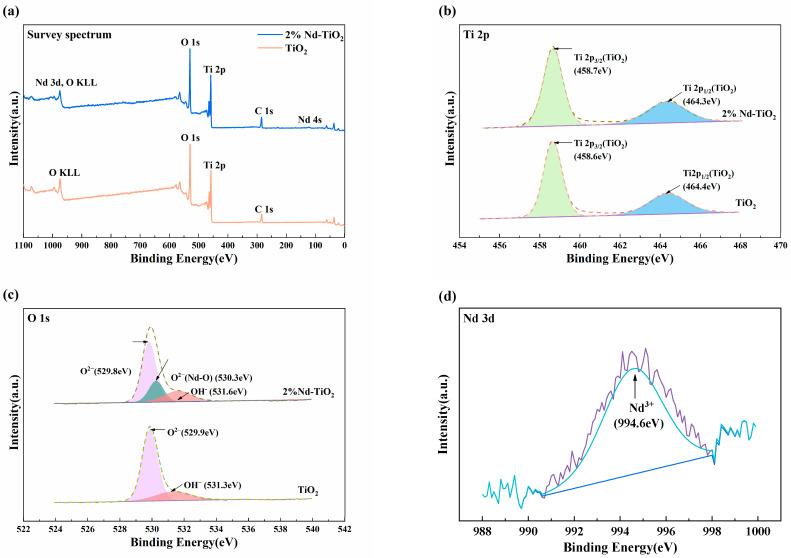
XPS spectra of 2%Nd-TiO_2_ and TiO_2_ NPs. (**a**) Survey spectrum; high-resolution XRS spectra of (**b**) Ti 2p, (**c**) O 1s, and (**d**) Nd 3d.

The chemical components at the surface of samples can be studied by the XPS technique. Figure 3a shows the XPS spectroscopic survey spectrum of 2%Nd-TiO_2_ and TiO_2_ NPs. XPS peaks showed that the samples contained C, Ti, O, and Nd elements (only C, Ti, and O for TiO_2_), corresponding to orbits of C 1s, Ti 2p, O 1s, and Nd 3d, respectively. Figure 3b shows the high-resolution XPS spectrum for Ti 2p of samples. There are two characteristic peaks located at 458.7 and 463.3 eV in Nd-TiO_2_, which correspond to Ti 2p_3/2_ and Ti 2p_1/2_ of TiO_2_, respectively, and the splitting value varies with the chemical components of samples and is about 5.7 eV. It indicates that the Ti element mainly exists as the chemical state of Ti^4+^. The O 1s region of TiO_2_ can be fitted by two peaks at 529.9 and 531.3 eV (Figure 3c), which are the Ti-O bond in TiO_2_ and hydroxyl groups. Additionally, three peaks of Nd-TiO_2_ are located at 529.8 eV, 530.3 eV, and 531.6 eV, which can be ascribed to Ti-O, Nd-O, and the hydroxyl group, respectively [38]. It can be confirmed that Nd^3+^ was doped in the TiO_2_ lattice. Figure 3d shows that the binding energy of Nd 3d is 994.6 eV. Although the peak intensity for Nd was weak, it can be ascribed to the presence of Nd in TiO_2_ [39,40,41].

### 3.4. UV–Vis DRS Analysis

**Figure 4:**
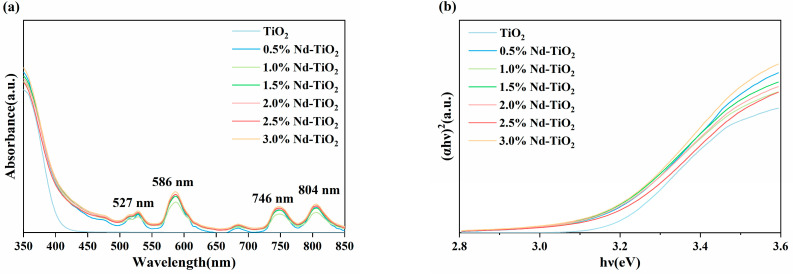
(**a**) UV–Vis absorption spectra of the TiO_2_ and Nd-TiO_2_ NPs with different Nd^3+^ concentrations; (**b**) relationship between (αhν)^2^ and photon energy (hν) for TiO_2_ and Nd-TiO_2_ NPs with different Nd^3+^ concentrations.

UV–Vis diffuse reflectance spectroscopy (DRS) is used to analyze the optical absorption properties under light irradiation in the wavelength range of 350–850 nm. The absorption spectra of TiO_2_ and Nd-TiO_2_ NPs are shown in Figure 4a. It is noteworthy that all samples have high optical absorption in the wavelength range below 400 nm, which can be attributed to the CT process between the O^2−^ and Ti^4+^, related to the electronic excitation from the valence band (VB) to the conduction band (CB) [42]. In addition, a redshift of the absorption edge toward the visible region was observed for all Nd-TiO_2_ samples compared with pure TiO_2_, which can be explained by the CT process between the TiO_2_ valence band and Nd^3+^ ion f electrons [43]. Moreover, pure TiO_2_ does not absorb in the visible light region (wavelength higher than 400 nm), but Nd-TiO_2_ samples exhibit outstanding absorption properties and have four characteristic absorption peaks at 527, 586, 762, and 804 nm, which are attributed to the 4f shell electron transition of Nd^3+^ from 4I_9/2_ ground to excited states ^2^K_13/2_ and ^4^G_7/2_, ^2^G_7/2_ and ^4^G_5/2_, ^4^S_3/2_ and ^4^F_7/2_, and ^4^F_5/2_ and ^2^H_9/2_, respectively [41]. The optical band gap (*E_g_*) of samples can be obtained by Tauc’s formula [44]:

$$\alpha h\nu =A{\left(h\nu -E_{g}\right)}^{\frac{1}{2}},$$

where *A* is a constant characteristic of the material, ν is frequency, α is absorption coefficient, and *h* stands for Planck’s constant. The bandgap is determined by plotting the relationship between (*αhν*)^2^ and photon energy (*hν*) and extrapolating the line to the *X*-axis. As shown in Figure 4b, the band gap values of TiO_2_ and Nd-TiO_2_ NPs were recorded as 3.20, 3.16, 3.15, 3.14, 3.14, 3.17, and 3.16 eV. The results show that Nd doping can narrow the bandgap of TiO_2_, but the bandgap shows less change with the increase in doping concentration. Nd doping can improve the optical absorption property and SERS performance of TiO_2_ NPs substrates, which is attributed to morphology, defects, and incorporation of impurities during synthesis in nanomaterials [29,32,45].

### 3.5. SERS Activity and Mechanism

**Figure 5:**
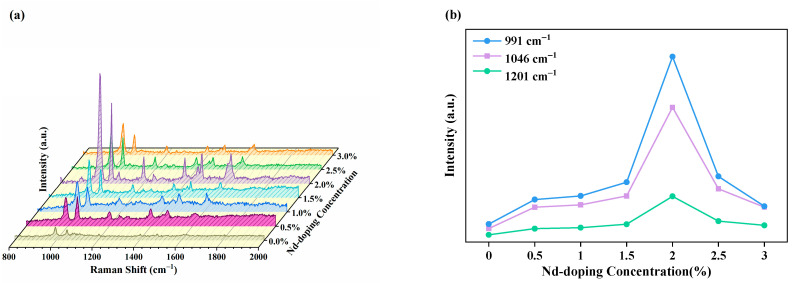
(**a**) SERS spectra of 4-Mpy (10^−2^ M) with different Nd doping concentrations; (**b**) Raman intensity for 4-Mpy with different Nd doping concentrations at 991, 1201, and 1046 cm^−1^ peaks.

To research the optimum doping concentration of SERS substrates, the Nd-TiO_2_ NPs (0%, 0.5%, 1%, 1.5%, 2%, 2.5%, and 3%) were measured with laser lines of 532 nm, 10^−^^2^ M 4-Mpy ethanol solution was used as probe molecules adsorbed on SERS substrates, as shown in Figure 5a, and the Raman peaks were located at 991, 1046, 1201, and 1614 cm^−^^1^. The strong bands at about 991 and 1046 cm^−^^1^ are assigned to ring breathing and pyridine ring C-H in-plane bending. Other weak bands at about 1201 and 1614 cm^−^^1^ are attributed to the CH deformation and NH stretching modes, and the pyridine ring C=C stretching mode. They are consistent with those previously reported for 4-Mpy on TiO_2_ NPs [46,47,48]. Figure 3b shows the relationship between the SERS intensities of the 991, 1046, and 1201 cm^−^^1^ bands of 4-Mpy and the Nd^3+^ concentration. The SERS signals are enhanced after Nd^3+^ doping and reach a maximum at 2% doping, which is attributed to the CT mechanism.

For the SERS substrates of semiconductor materials, the frequency of LSPR is located in the infrared region, far away from the 532 nm laser source [49]. It can be calculated as follows:

$$\omega_{p}={\left(\frac{4\pi ne^{2}}{\varepsilon_{\infty }m_{e}}\right)}^{\frac{1}{2}}$$

where *n* is the electron transmission density, and *m_e_* is the electron mass. Therefore, it can be judged that the chemical enhancement mechanism plays a major role in SERS enhancement for this system. Chemical enhancement is primarily due to the charge-transfer between the probe molecule and semiconductor SERS substrates.

**Figure 6:**
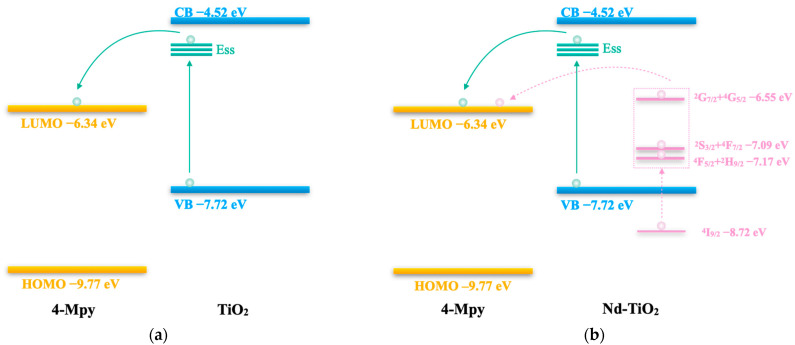
Schematic diagram of the charge-transfer (CT) mechanism for SERS between (**a**) 4-Mpy and TiO_2_ and (**b**) 4-Mpy and Nd-TiO_2_.

The CT mechanism for SERS of TiO_2_ and Nd-TiO_2_ NPs with 4-Mpy is illustrated in Figure 6. From the previous literature, the lowest unoccupied molecular orbital (LUMO) levels and the highest occupied molecular orbital (HOMO) of 4-Mpy are −9.77 and −6.34 eV, respectively [50]. The minimum conduction band (CB) and maximum valence band (VB) of TiO_2_ are −7.72 and −4.52 eV, respectively [33]. Ess is the surface state energy level, which is generated by the binding of the electron at surface defects (such as surface oxygen vacancies) of TiO_2_ NPs, and Ess is located at about 0.5 eV below the CB [51].

As shown in Figure 6a, with a 532 nm (ca. 2.33 eV) laser, the electrons can be excited from VB of TiO_2_ to the Ess, then transferred to the LUMO of 4-Mpy, and return to the CB of TiO_2_, to release Raman scattered photons. As the pure anatase TiO_2_ SERS substrate has only a few oxygen vacancies, the SERS intensity is very low. Ess acts as an intermediary in the CT transition of TiO_2_ to 4-Mpy. As shown in Figure 6b, through Nd doping, (1) Nd^3+^ will diffuse into the TiO_2_ lattice to replace part of the Ti^4+^, narrow the bandgap, and generate more oxygen vacancies, thereby increasing the concentration of defects of TiO_2_ (more Ess generated), so that increasing numbers of Ess can promote the CT process of TiO_2_ to 4-Mpy and enhance the SERS signal; (2) in addition, the characteristic energy level of Nd^3+^ can be introduced into the bandgap of TiO_2_ by doping, and the locations of the electronic ground state (^4^I_9/2_) and excited state energy levels (^4^F_5/2_+^2^H_9/2_, ^4^S_3/2_+^4^F_7/2_, and ^2^G_7/2_+^4^G_5/2_) of Nd^3+^ can be calculated with the Dorenbos model [33,52,53], which are at −8.72, −7.17, −7.09, and −6.55 eV, respectively. With a 532 nm laser, the 4f electrons of Nd^3+^ can be excited from the ground state (^4^I_9/2_) to the excited states (^4^F_5/2_+^2^H_9/2_, ^4^S_3/2_+^4^F_7/2_, and ^2^G_7/2_+^4^G_5/2_), then transferred to the LUMO of 4-Mpy, and return to the ground state of the Nd^3+^, to release Raman scattered photons. The above two CT processes after Nd doping work together to enhance the SERS signal and eliminate the fluorescent background.

**Figure 7:**
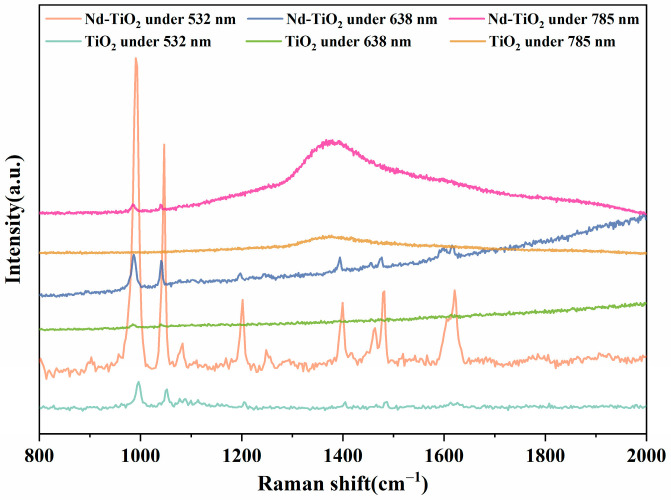
SERS spectra of 4-Mpy adsorbed on Nd-TiO_2_ and TiO_2_ NPs under 532, 638, and 785 nm laser excitation.

The CT process between Nd-TiO_2_ and 4-Mpy was further validated by SERS analysis using 638 and 785 nm laser wavelengths. As shown in Figure 7, the SERS signal intensity was dramatically reduced with a certain degree of background fluorescence (the intensity curves show an upward trend) with 638 nm (ca. 1.94 eV) laser irradiation, which is due to the inability of the laser energy to transfer the electrons from the ground state (^4^I_9/2_) to the excited state (^2^G_7/2_ + ^4^G_5/2_). However, under the irradiation of the 785 nm laser (ca. 1.58 eV), the SERS signal intensity decreased significantly and was accompanied by a strong background fluorescence signal, and the characteristic peak of 4-Mpy could not be observed on the TiO_2_ substrate, which may be because the laser energy at 785 nm can only transfer the electrons from the ground state (^4^I_9/2_) to the excited state (^4^F_5/2_ + ^2^H_9/2_). The above two SERS analyses under 633 and 785 nm lasers are sufficient to verify the unique CT process of this work. Meanwhile, 2% Nd is used as the optimum doping concentration for TiO_2_ NPs SERS substrates since high levels of defects caused by high doping concentrations lead to the recombination of electron–holes and bind electrons for the CT process of Nd-TiO_2_ to 4-Mpy [54].

### 3.6. SERS Performance

**Figure 8:**
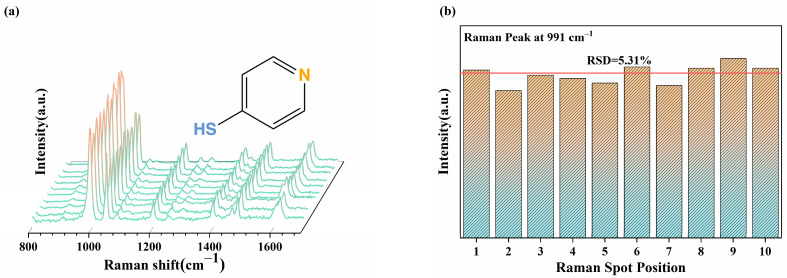
(**a**) SERS spectra of 10^−^^2^ M 4-Mpy acquired from 10 different spots on the 2%Nd-TiO_2_ NPs substrate; (**b**) Raman intensity distribution at 991 cm^−^^1^ from (**a**) for RSD calculation (the average peak intensity is indicated by the red line).

To evaluate the reproducibility of the Nd-TiO_2_ SERS substrates, the SERS spectra of 4-Mpy were acquired from ten different spots on the substrate. As shown in Figure 8a, the intensity of the main characteristic peaks at 991 cm^−^^1^ showed high consistency. Figure 8b gives information about the relative standard deviation (RSD) of the peak intensity at 991 cm^−1^. The RSD is about 5.31%, which indicates excellent reproducibility of the SERS substrates for practical applications.

**Figure 9:**
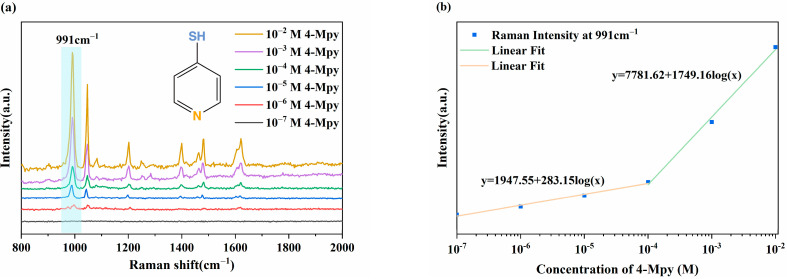
(**a**) SERS spectra of the 4-MPy on 2%Nd-TiO_2_ NPs substrates with different concentrations from 10^−2^ to 10^−7^ M; (**b**) Raman intensity at 991 cm^−1^ versus the concentration of 4-Mpy ranging from 10^−2^ to 10^−7^ M.

To analyze the SERS sensitivity and the limit of detection (LOD) of the Nd-TiO_2_ NPs substrates, the SERS spectra of 4-Mpy with different concentrations (10^−^^2^ to 10^−^^7^ M, ethanol solutions) were recorded and are shown in Figure 9a. It can be seen that the SERS intensity of the main characteristic peaks decreased as the concentration decreases. The correlation between Raman intensity at 991 cm^−^^1^ and the concentration of 4-Mpy are listed in Figure 9b. The linear relationship equations are y = 1947.55 + 283.15log(x) in the concentration range 10^−^^7^ to 10^−^^4^ M and y = 7781.62 + 1749.16log(x) in the concentration range from 10^−^^4^ to 10^−^^2^ M. The correlation coefficients (R^2^) are 0.982 and 0.992, respectively. It can be seen that at a low concentration range, the Raman signal intensity changes drastically as the concentration of the probe molecule changes, while in a relatively high concentration region, its intensity changes slowly (the logarithm values of concentration were set as the X-axis so that the slopes in the equation in the low-concentration region were smaller than those in the high-concentration region). This linear phenomenon has also been reported in Qin and Cheng’s previous works [55,56], and is due to the multi-layer adsorption of probe molecules at high concentrations, which weaken the enhancement effect. However, at low concentrations, the probe molecules will directly act on the surface of the SERS substrate, resulting in a significant enhancement with a high correlation coefficient. The LOD was calculated as 10^−^^6^ M, which is much lower than other semiconductor SERS substrates. These results show excellent SERS sensitivity and ultra-low LOD on the Nd-TiO_2_ substrates.

To quantitatively investigate the enhancement ability, the enhancement factor (EF) for as-prepared SERS substrate can be calculated by the following equation:

$$EF=\left(\frac{I_{SERS}}{I_{Bulk}}\right)\left(\frac{N_{Bulk}}{N_{SERS}}\right)$$

where *I_SERS_* and *I_Bulk_* are the Raman intensities of 4-Mpy in the SERS spectra and standard Raman spectra and *N_SERS_* and *N_Raman_* present the numbers of 4-Mpy molecules adsorbed on the SERS substrate and in the bulk condition, respectively.

The ratio of intensities (*I_SERS_*/*I_Bulk_*) can be obtained by 4-Mpy in the SERS spectra and standard Raman spectra (Figure S2), and is 37.57. The *N_Bulk_* and *N_SERS_* are 7.83 × 10^10^ and 7.79 × 10^7^, respectively (the detailed calculation process is shown in the Supporting Information). Therefore, the EF of Nd-TiO_2_ NPs SERS substrate at the main characteristic Raman band of 991 cm^−^^1^ can reach about 3.79 × 10^4^, which is higher than the other reported semiconductor SERS substrates [32,57].

## 4. Conclusions

In conclusion, Nd-TiO_2_ NPs were synthesized with different atomic ratios of Nd by the sol–gel method and 4-Mpy was used as a probe molecule to investigate its SERS properties and the CT process in chemical enhancement mechanisms. The results indicated that the enhanced intensities of SERS signals by Nd^3+^ doping could be summarized as, on the one hand, increased defect concentrations promoting the CT process between TiO_2_ and 4-Mpy while, on the other hand, the introduction the 4f orbital energy level of Nd^3+^ created a unique CT process between Nd^3+^ and 4-Mpy. Moreover, it is innovative to use a rare-earth element (Nd) and the Dorenbos model to study the CT mechanism of SERS. The SERS analysis was validated under different excitation wavelengths. Meanwhile, the SERS substrate has high reproducibility of SERS signals, and the LOD can be expanded to 10^−^^6^ M with the EF to 3.79 × 10^4^, which is superior to other semiconductor SERS substrates. This work provides new ideas for the selective detection of different probe molecules, which will attract more attention in the field of application.

## Data Availability

The data presented in this study are available on request from the corresponding author. The data are not publicly available due to funder retention policies.

## Supplementary Materials

The following are available online at https://www.mdpi.com/article/10.3390/nano11082063/s1, Figure S1: Characterization analysis of the TiO_2_ NPs. (a) SEM and (b) TEM images; (c) HRTEM image showing lattice space; (d) SAED pattern mainly showing the (101), (004), (200), (105), and (213) crystal faces; the element mapping of (e) O and (f) Ti; (g) EDS spectrum, Figure S2: SERS spectra of 4-Mpy on Nd-TiO_2_ NPs substrate (blue line) and in bulk condition (green line).

## References

[1] Zhao Y.-X., Zhu W.-W., Wu Y.-Y., Chen Y.-Y., Du F.-K., Yan J., Tan X.-C., Wang Q. (2021). Sensitive surface-enhanced Raman scattering for the quantitative detection of formaldehyde in foods using gold nanorod substrate. Microchem. J..

[2] Zhou J., Sheth S., Zhou H., Song Q. (2020). Highly selective detection of l-Phenylalanine by molecularly imprinted polymers coated Au nanoparticles via surface-enhanced Raman scattering. Talanta.

[3] Zheng Z., Cong S., Gong W., Xuan J., Li G., Lu W., Geng F., Zhao Z. (2017). Semiconductor SERS enhancement enabled by oxygen incorporation. Nat. Commun..

[4] Lee J.-H., Nam J.-M., Jeon K.-S., Lim D.-K., Kim H., Kwon S., Lee H., Suh Y.D. (2012). Tuning and Maximizing the Single-Molecule Surface-Enhanced Raman Scattering from DNA-Tethered Nanodumbbells. ACS Nano.

[5] Su D., Jiang S., Yu M., Zhang G., Liu H., Li M.-Y. (2018). Facile fabrication of configuration controllable self-assembled Al nanostructures as UV SERS substrates. Nanoscale.

[6] Li X., Choy W.C.H., Ren X., Zhang D., Lu H. (2014). Highly intensified surface enhanced Raman scattering by using monolayer graphene as the nanospacer of metal film-metal nanoparticle coupling system. Adv. Funct Mater..

[7] Kong X.-K., Chen Q.-W., Sun Z.-Y. (2013). Enhanced SERS of the complex substrate using Au supported on graphene with pyridine and R6G as the probe molecules. Chem. Phys. Lett..

[8] Wu H.Y., Choi C.J., Cunningham B.T. (2012). Plasmonic nanogap-enhanced raman scattering using a resonant nanodome array. Small.

[9] Kim K., Lee H.B., Yoon J.K., Shin D., Shin K.S. (2010). Ag nanoparticle-mediated Raman scattering of 4-aminobenzenethiol on a Pt substrate. J. Phys. Chem. C.

[10] McMahon J.M., Henry A.I., Wustholz K.L., Natan M.J., Freeman R.G., Van Duyne R.P., Schatz G.C. (2009). Gold nanoparticle dimer plasmonics: Finite element method calculations of the electromagnetic enhancement to surface-enhanced Raman spectroscopy. Anal. Bioanal. Chem..

[11] Chen L., Zhang F., Deng X.Y., Xue X., Wang L., Sun Y., Feng J.D., Zhang Y., Wang Y., Jung Y.M. (2018). SERS study of surface plasmon resonance induced carrier movement in Au@Cu_2_O core-shell nanoparticles. Spectrochim. Acta Part. A Mol. Biomol. Spectrosc..

[12] Morton S.M., Jensen L. (2009). Understanding the molecule-surface chemical coupling in SERS. J. Am. Chem. Soc..

[13] Chen R., Jensen L. (2020). Interpreting the chemical mechanism in SERS using a Raman bond model. J. Chem. Phys..

[14] Park W.H., Kim Z.H. (2010). Charge transfer enhancement in the SERS of a single molecule. Nano Lett..

[15] Moore J.E., Morton S.M., Jensen L. (2012). Importance of correctly describing charge-transfer excitations for understanding the chemical effect in SERS. J. Phys. Chem. Lett..

[16] Kneipp K. (2016). Chemical Contribution to SERS Enhancement: An Experimental Study on a Series of Polymethine Dyes on Silver Nanoaggregates. J. Phys. Chem. C.

[17] Mao Z., Song W., Xue X., Ji W., Li Z., Chen L., Mao H., Lv H., Wang X., Lombardi J.R. (2012). Interfacial charge-transfer effects in semiconductor-molecule-metal structures: Influence of contact variation. J. Phys. Chem. C.

[18] Kudelski A., Bukowska J. (1996). The chemical effect in surface enhanced Raman scattering (SERS) for piperidine adsorbed on a silver electrode. Surf. Sci..

[19] Zheng X., Ren F., Zhang S., Zhang X., Wu H., Zhang X., Xing Z., Qin W., Liu Y., Jiang C. (2017). A General Method for Large-Scale Fabrication of Semiconducting Oxides with High SERS Sensitivity. ACS Appl. Mater. Interfaces.

[20] Wang X.T., Shi W.S., She G.W., Mu L.X. (2012). Surface-Enhanced Raman Scattering (SERS) on transition metal and semiconductor nanostructures. Phys. Chem. Chem. Phys..

[21] Zhang M., Sun H., Chen X., Zhou H., Xiong L., Chen W., Chen Z., Bao Z., Wu Y. (2021). The influences of graphene oxide (GO) and plasmonic Ag nanoparticles modification on the SERS sensing performance of TiO_2_ nanosheet arrays. J. Alloy. Compd..

[22] Qiu H., Wang M., Cao M., Zhang L., Ji S., Dou J., Ji Y., Kou S., Guo J., Yang Z. (2019). Self-cleaning SERS membrane for reusable and ultrasensitive molecular detection via integrating graphitic-carbon-nitride nanosheets and Ag nanospheres into hierarchical graphene layers that covered with graphitic-carbon-nitride quantum-dots. Appl. Surf. Sci..

[23] Ghopry S.A., Alamri M.A., Goul R., Sakidja R., Wu J.Z. (2019). Extraordinary Sensitivity of Surface-Enhanced Raman Spectroscopy of Molecules on MoS_2_ (WS_2_) Nanodomes/Graphene van der Waals Heterostructure Substrates. Adv. Opt. Mater..

[24] Zhao Y.C., Zhang Q.J., Ma L.P., Song P., Xia L.X. (2020). A P/N type silicon semiconductor loaded with silver nanoparticles used as a SERS substrate to selectively drive the coupling reaction induced by surface plasmons. Nanoscale Adv..

[25] Yamada H., Yamamoto Y., Tani N. (1982). Surface-enhanced raman scattering (SERS) of adsorbed molecules on smooth surfaces of metals and a metal oxide. Chem. Phys. Lett..

[26] Yang L., Jiang X., Ruan W., Zhao B., Xu W., Lombardi J.R. (2008). Observation of Enhanced Raman Scattering for Molecules Adsorbed on TiO_2_ Nanoparticles: Charge-Transfer Contribution. J. Phys. Chem. C.

[27] Xue X., Ji W., Mao Z., Mao H., Wang Y., Wang X., Ruan W., Zhao B., Lombardi J.R. (2012). Raman Investigation of Nanosized TiO_2_: Effect of Crystallite Size and Quantum Confinement. J. Phys. Chem. C.

[28] Wang Y., Ruan W., Zhang J., Bai Y., Lombardi J.R. (2010). Direct observation of surface-enhanced Raman scattering in ZnO nanocrystals. J. Raman Spectrosc..

[29] Zhou L., Zhou J., Lai W., Yang X., Meng J., Su L., Gu C., Jiang T., Pun E.Y.B., Shao L. (2020). Irreversible accumulated SERS behavior of the molecule-linked silver and silver-doped titanium dioxide hybrid system. Nat. Commun..

[30] Tian Y., Wei H., Xu Y., Sun Q., Man B., Liu M. (2020). Influence of sers activity of SnSe_2_ nanosheets doped with sulfur. Nanomaterials.

[31] Yang L., Peng Y., Yang Y., Liu J., Huang H., Yu B., Zhao J., Lu Y., Huang Z., Li Z. (2019). A Novel Ultra-Sensitive Semiconductor SERS Substrate Boosted by the Coupled Resonance Effect. Adv. Sci..

[32] Li P., Wang X., Zhang X., Zhang L., Yang X., Zhao B. (2019). Investigation of the charge-transfer between Ga-doped ZnO nanoparticles and molecules using surface-enhanced Raman scattering: Doping induced band-gap shrinkage. Front. Chem..

[33] Dorenbos P. (2013). The Electronic Structure of Lanthanide Impurities in TiO_2_, ZnO, SnO_2_, and Related Compounds. ECS J. Solid State Sci. Technol..

[34] Serga V., Burve R., Krumina A., Romanova M., Kotomin E.A., Popov A.I. (2021). Extraction–Pyrolytic Method for TiO_2_ Polymorphs Production. Crystals.

[35] Serga V., Burve R., Krumina A., Pankratova V., Popov A.I., Pankratov V. (2021). Study of phase composition, photocatalytic activity, and photoluminescence of TiO_2_ with Eu additive produced by the extraction-pyrolytic method. J. Mater. Res. Technol..

[36] Gupta S.K., Desai R., Jha P.K., Sahoo S., Kirin D. (2010). Titanium dioxide synthesized using titanium chloride: Size effect study using Raman spectroscopy and photoluminescence. J. Raman Spectrosc..

[37] Wu X., Yin S., Dong Q., Guo C., Kimura T., Matsushita J.-i., Sato T. (2013). Photocatalytic Properties of Nd and C Codoped TiO_2_ with the Whole Range of Visible Light Absorption. J. Phys. Chem. C.

[38] Hewer T., Souza E., Martins T., Muccillo E., Freire R. (2011). Influence of neodymium ions on photocatalytic activity of TiO_2_ synthesized by sol–gel and precipitation methods. J. Mol. Catal. A Chem..

[39] Gaya U.I., Abdullah A.H. (2008). Heterogeneous photocatalytic degradation of organic contaminants over titanium dioxide: A review of fundamentals, progress and problems. J. Photochem. Photobiol. C Photochem. Rev..

[40] Bao R., Yu Y., Chen H., Wang W., Xia J., Li H. (2018). Effects of Rare Earth Elements and Nitrogen Co-Doped on the Photocatalytic Performance of TiO_2_. Cryst. Res. Technol..

[41] Yuan M., Zhang J., Yan S., Luo G., Xu Q., Wang X., Li C. (2011). Effect of Nd_2_O_3_ addition on the surface phase of TiO_2_ and photocatalytic activity studied by UV Raman spectroscopy. J. Alloy. Compd..

[42] Jing L., Xin B., Yuan F., Xue L., Wang B., Fu H. (2006). Effects of Surface Oxygen Vacancies on Photophysical and Photochemical Processes of Zn-Doped TiO_2_ Nanoparticles and Their Relationships. J. Phys. Chem. B.

[43] Borgarello E., Kiwi J., Graetzel M., Pelizzetti E., Visca M. (1982). Visible light induced water cleavage in colloidal solutions of chromium-doped titanium dioxide particles. J. Am. Chem. Soc..

[44] Tauc J. (1968). Optical properties and electronic structure of amorphous Ge and Si. Mater. Res. Bull..

[45] Nithyaa N., Victor Jaya N. (2021). Effect of Nd on structural, optical and magnetic behaviour of TiO_2_ nanoparticles. Appl. Phys. A.

[46] Song W., Wang Y., Zhao B. (2007). Surface-enhanced Raman scattering of 4-mercaptopyridine on the surface of TiO_2_ nanofibers coated with Ag nanoparticles. J. Phys. Chem. C.

[47] Wang Y., Sun Z., Hu H., Jing S., Zhao B., Xu W., Zhao C., Lombardi J.R. (2007). Raman scattering study of molecules adsorbed on ZnS nanocrystals. J. Raman Spectrosc..

[48] Baldwin J., Schühler N., Butler I.S., Andrews M.P. (1996). Integrated optics evanescent wave surface enhanced Raman scattering (IO-EWSERS) of mercaptopyridines on a planar optical chemical bench: Binding of hydrogen and copper ion. Langmuir.

[49] Kittel C., McEuen P., McEuen P. (1996). Introduction to Solid State Physics.

[50] Yang S., Yao J., Quan Y., Hu M., Su R., Gao M., Han D., Yang J. (2020). Monitoring the charge-transfer process in a Nd-doped semiconductor based on photoluminescence and SERS technology. Light Sci. Appl..

[51] Boschloo G., Fitzmaurice D. (1999). Spectroelectrochemical investigation of surface states in nanostructured TiO_2_ electrodes. J. Phys. Chem. B.

[52] Dorenbos P. (2005). Valence stability of lanthanide ions in inorganic compounds. Chem. Mater..

[53] Dorenbos P. (2017). Charge transfer bands in optical materials and related defect level location. Opt. Mater..

[54] Xue X., Ruan W., Yang L., Ji W., Xie Y., Chen L., Song W., Zhao B., Lombardi J.R. (2012). Surface-enhanced Raman scattering of molecules adsorbed on Co-doped ZnO nanoparticles. J. Raman Spectrosc..

[55] Chen J., Qin G., Shen W., Li Y., Das B. (2015). Fabrication of long-range ordered, broccoli-like SERS arrays and application in detecting endocrine disrupting chemicals. J. Mater. Chem. C.

[56] Cheng H.-W., Huan S.-Y., Wu H.-L., Shen G.-L., Yu R.-Q. (2009). Surface-enhanced Raman spectroscopic detection of a bacteria biomarker using gold nanoparticle immobilized substrates. Anal. Chem..

[57] Qi D., Lu L., Wang L., Zhang J. (2014). Improved SERS sensitivity on plasmon-free TiO_2_ photonic microarray by enhancing light-matter coupling. J. Am. Chem. Soc..

