# Supplementary material for: Charge-Transfer Process in Surface-Enhanced Raman Scattering Based on Energy Level Locations of Rare-Earth Nd3+-Doped TiO2 Nanoparticles
Source: Nanomaterials (Basel). 2021 Aug 14;11(8):2063. doi: 10.3390/nano11082063 (PMC8400391; doi:10.3390/nano11082063)
Supplement: Supplementary file 1 [file nanomaterials-11-02063-s001.zip › nanomaterials-1331851-supplementary.pdf]

Supplementary Files

Charge-Transfer Process in Surface-Enhanced Raman Scattering Based on Energy Level Locations of Rare-Earth Nd^{3+}-Doped TiO_2 Nanoparticles

Zihao Zhao [1], Xiang Zhao [1,2,*], Mu Zhang [1,2] and Xudong Sun [2,3,*]

[1] Key Laboratory for Anisotropy and Texture of Materials (Ministry of Education), Northeastern University, Shenyang 110819, China; 1970495@stu.neu.edu.cn (Z.Z.); zhangm@mail.neu.edu.cn (M.Z.)
[2] Lab. of Advanced Ceramics, Foshan Graduate School, Northeastern University, Foshan 528311, China
[3] State Key Laboratory of Rolling and Automation, Northeastern University, Shenyang 110819, China
* Correspondence: 1310142@stu.neu.edu.cn (X.Z.); xdsun@mail.neu.edu.cn (X.S.)

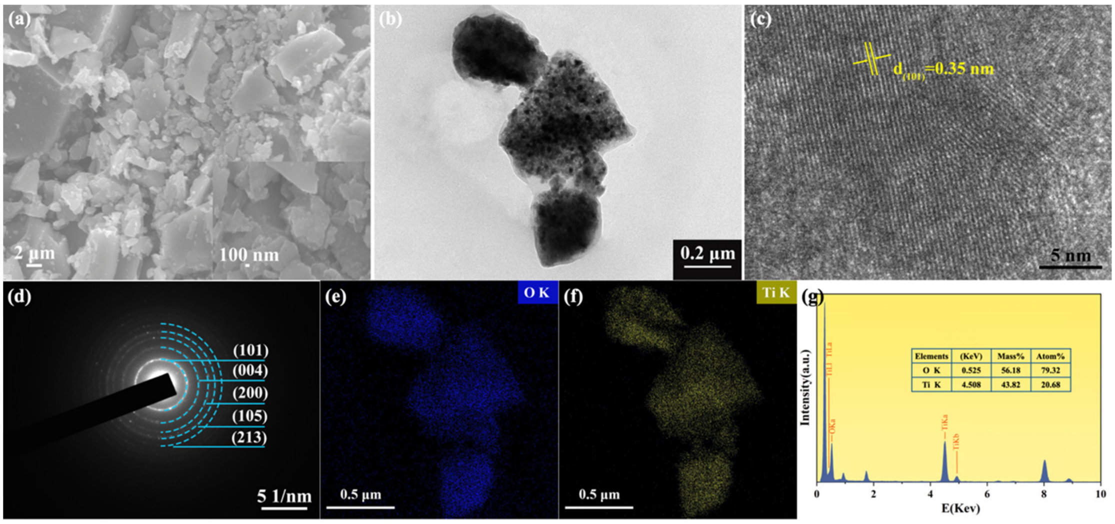

Figure **S1**: Characterization analysis of the TiO_2 NPs. (**a**) SEM and (**b**) TEM images; (**c**) HRTEM image showing lattice space; (**d**) SAED pattern mainly showing the (101), (004), (200), (105) ,and (213) crystal faces; the element mapping of (**e**) O, (**f**) Ti, respectively; (**g**) EDS spectrum.

Figure S1 shows the characterization analysis of the TiO_2 NPs by SEM and TEM. As shown in Figures S1a and b, it can be seen that the TiO_2 NPs are agglomerated in sharp and irregular block shapes. The HRTEM image of TiO_2 also shows clear lattice fringes and 0.35 nm interplanar crystal space. These are similar to the Nd-doped samples, indicating that the small amount of doping has little effect on the morphology and crystal structure of the TiO_2 NPs. In the SAED pattern (Figure S2d), TiO_2 NPs are polycrystalline structures of anatase TiO_2 with (101), (004), (200), (105), and (213) concentric diffraction rings, but the diffraction rings of the doped samples are much clearer. The elemental mapping patterns (Figure S2 e and f) and EDS show that the distribution and amount of O and Ti element and unmarked peaks are copper

ions from the copper grid of the sample holder.

Calculation of enhancement factor (EF)

To quantitative investigate the enhancement ability, the enhancement factor (EF) for as prepared SERS substrate can be calculated by the following equation:

$$EF = (\frac{I_{SERS}}{I_{Bulk}})(\frac{N_{Bulk}}{N_{SERS}})$$

where I_{SERS} and I_{Bulk} are the Raman intensities of 4-Mpy in the SERS spectra and standard Raman spectra; Where N_{SERS} and N_{Raman} present the numbers of 4-Mpy molecules adsorbed on the SERS substrate and in the bulk condition, respectively.

The ratio of intensities (I_{SERS}/I_{Bulk}) can be obtained by 4-Mpy in the SERS spectra and standard Raman spectra (Figure.S2), which is 37.57

The N_{Bulk} is related to the density of the sample (ρ = 1.2 g ·cm^{-3}) and the volume of laser irradiation[1]. The volume of laser irradiation can be calculated from 532 laser excitation focus depths (h = 21 μm) and the diameter of laser beam spot (D = 0.72 μm). So, the mass of the 4-Mpy in bulk condition within laser irradiation is 1.03×10^{-11} g. Moreover, the N_{Bulk} can be calculated from the known molar mass of 4-Mpy (79.1 g ·mol^{-1}) and Avogadro's constant, which is 7.83×10^{10}.

The N_{SERS} can be estimated by concentration and volume of 4-Mpy adsorbed on Nd-TiO_2 NPs (10^{-2} M and 10 μL). The amount of molecules is 1×10^{-7} mol and spreads onto a glass slide of 3.14 cm^2. The number density of molecules on the surface is 1.91×10^8 molecules ·μm^{-2}. Thus N_{SERS} = 7.79×10^7 on the 0.72 um diameter laser[2]. Therefore, the EF = (I_{SERS}/I_{bulk}) × (N_{Bulk}/N_{SERS}) =3.79×10^4.

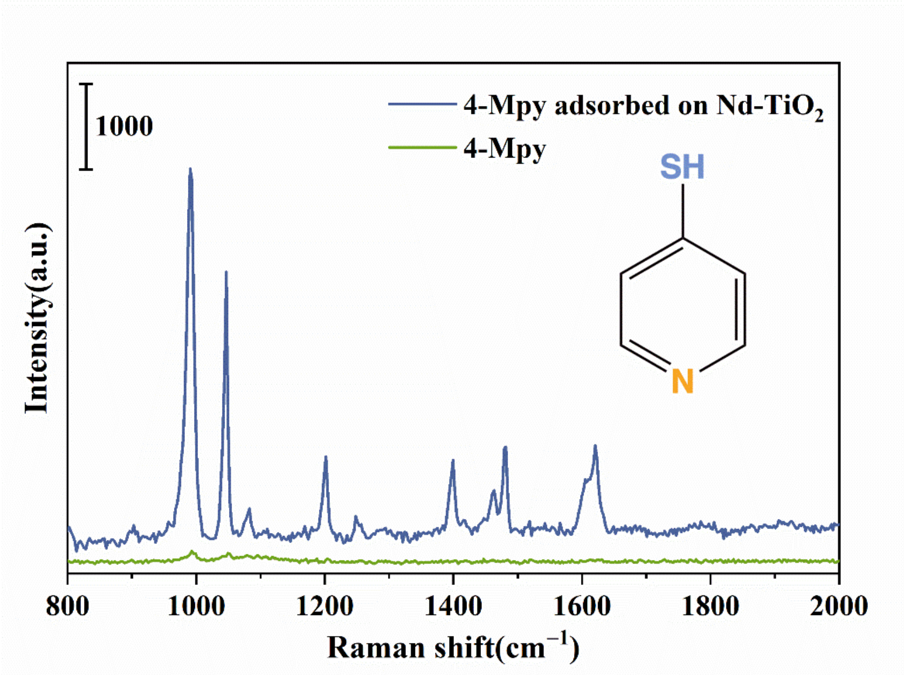

Figure **S2**. SERS spectra of 4-Mpy on Nd-TiO_2 NPs substrate (blue line) and in bulk condition (green line).

References

1. Li, M.; Gao, Y.; Fan, X.; Wei, Y.; Hao, Q.; Qiu, T. Origin of layer-dependent SERS tunability in 2D transition metal dichalcogenides. *Nanoscale Horizons* **2021**, *6*, 186-191.
2. Park, H.K.; Yoon, J.K.; Kim, K. Novel fabrication of Ag thin film on glass for efficient surface-enhanced Raman scattering. *Langmuir* **2006**, *22*, 1626-1629.
